# Deep Vein Thrombosis Secondary to Extrinsic Compression: A Case Report

**DOI:** 10.7759/cureus.11160

**Published:** 2020-10-25

**Authors:** Gurdeep Singh, Sanad Alshareef, Murali Meka

**Affiliations:** 1 Internal Medicine, Cape Fear Valley Medical Center, Fayetteville, USA; 2 Interventional Radiology, Cape Fear Valley Medical Center, Fayetteville, USA

**Keywords:** deep vein thrombosis (dvt), endovascular stenting, may-thurner's syndrome, endovascular catheter-directed thrombolysis, interventional radiology

## Abstract

May-Thurner syndrome (MTS) is defined as extrinsic venous compression by the arteries of the iliocaval system. The most common manifestation of MTS is compression of the left common iliac vein by the right common iliac artery.

May-Thurner syndrome is pathologically seen in 2%-5% of patients presenting with symptomatic deep vein thrombosis (DVT). As an anatomic variant, the prevalence is predicted to be much higher as most patients with MTS anatomy are asymptomatic and do not seek evaluation.

Symptomatic clinical presentations can include left lower extremity pain, swelling, skin discoloration, ulceration, and venous claudication. Here we present a patient with symptomatic MTS.

A 64-year-old female with no pertinent past medical history presented with complaint of worsening left lower extremity swelling and pain. Clinical picture was concerning for phlegmasia cerulea dolens and ultrasound was bypassed in favor of a CT scan of the lower extremities bilaterally.

The CT showed occlusion of the deep veins of the left leg secondary to stenosis of the left common iliac vein just posterior to the right common iliac artery; a finding consistent with MTS. Interventional radiology performed a catheter-directed thrombolysis with stenting of the left common iliac and external iliac veins. The patient clinically improved and was discharged with anticoagulative therapy.

May-Thurner syndrome is a condition that typically manifests due to external anatomic compression of the left common iliac vein. It is our belief that patients (with the appropriate risk factors) presenting with signs and symptoms consistent with proximal lower extremity DVT would benefit from further radiographic studies to fully evaluate for iliocaval venous stenosis and subsequent catheter-directed thrombolysis with endovascular stenting.

## Introduction

May-Thurner syndrome (MTS) is a pathologically variable condition characterized by extrinsic venous compression of the iliocaval vessels, leading to a varying degree of venous outflow obstruction. It is also commonly referred to as iliocaval venous compression syndrome, iliac vein compression syndrome, or Cockett's syndrome. While anatomic variants have been discovered, the most common manifestation of MTS is compression of the left common iliac vein between the right common iliac artery and the fifth lumbar vertebrae [1].

It has been known since the mid-1800s that deep vein thrombosis (DVT) was about five times more likely to occur in the left lower extremity than the right lower extremity but the etiology behind this was never properly elucidated. Roughly 100 years later, May and Thurner examined 430 cadavers and ultimately discovered intraluminal thickening within the left common iliac vein in roughly 22% of these cadavers [2]. They termed this thickening "venous spurs" and deemed their presence to be directly related to the pulsatile waves from the right common iliac artery pressing the vein against the fifth lumbar vertebrae. Seventeen years after that, Cockett et al. illustrated that this anatomical variant can predispose patients to the development of chronic thromboses [3].

The risk factors for MTS include female gender (especially those taking oral contraceptives or are multiparous), scoliosis, dehydration, hypercoagulable states, and cumulative radiation exposure [1]. MTS as a pathologic variant is seen in 2%-5% of patients presenting with symptomatic DVTs [1]. As an anatomic variant, the prevalence is predicted to be much higher as most patients with MTS anatomy do not seek evaluation until they become symptomatic [1]. While the majority of individuals with MTS are asymptomatic, clinical presentations can include left lower extremity pain, swelling, acute DVT, skin discoloration, ulceration, and venous claudication [1]. Here, we present a patient with symptomatic MTS to illustrate the proper approach, management, and treatment of this condition. Discussion on treatment specifically will be focused on juxtaposing current surgical and medical therapies while also addressing the importance of preventing complications such as post-thrombotic syndrome.

## Case presentation

A 64-year-old female presented to the ED with complaint of acute left lower extremity pain with associated swelling and discoloration. Symptoms initially presented the day prior to admission in her left groin area and had worsened overnight. The patient described the pain as “achy” and endorsed difficulty with ambulation but denied any trauma or injury to the extremity. Also denied any fever, chills, chest pain, shortness of breath, or dyspnea on exertion. She was initially evaluated at an outside facility, where she was found to have an elevated D-Dimer but given the lack of ultrasound technology, she was subsequently transferred over after receiving 90 mg lovenox.

**Figure 1:**
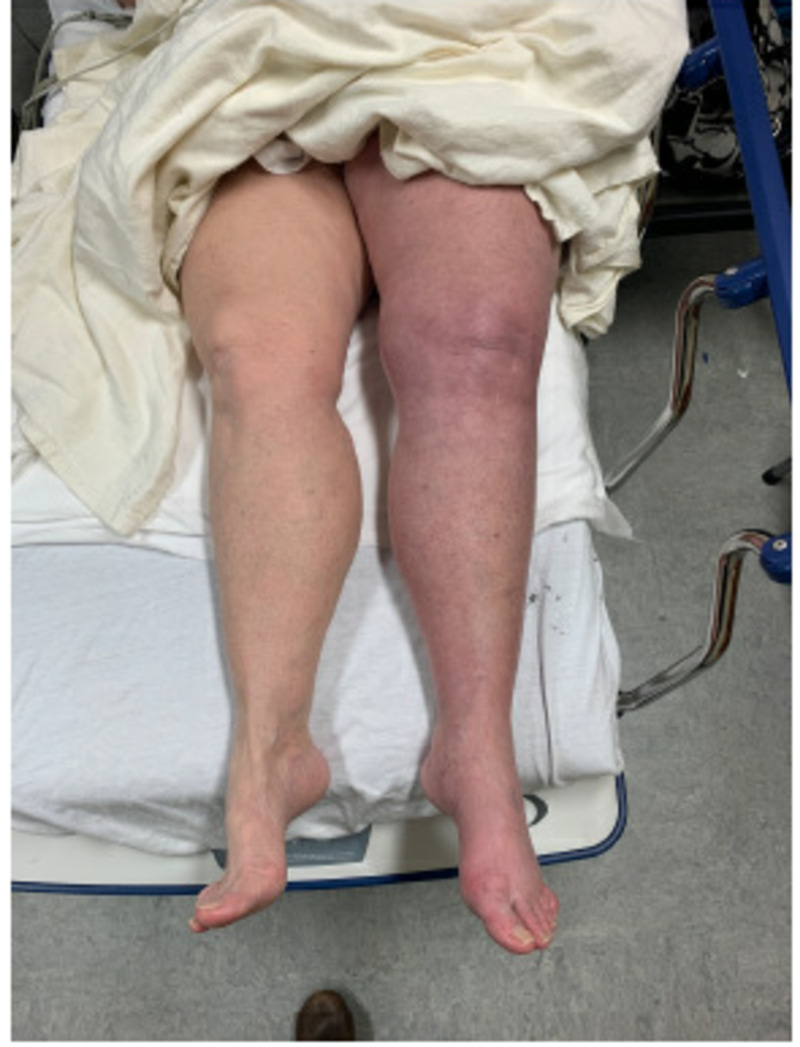
Initial presentation.

The patient’s medical history was noncontributory. She denied any history of prior DVT, pulmonary embolism, malignancy, hormone replacement therapy, recent surgery, or period of prolonged immobilization. She formerly smoked cigarettes and admitted to smoking E-cigarettes on an intermittent basis. The patient admits to having a family history of von Willebrand’s disease but states she tested negative in the past. The patient endorsed being allergic to percocet but denied having any other medical allergies. She also denied taking any scheduled medications. Review of systems was only positive for left leg swelling, left leg pain, and color change (Figure 1).

Physical exam was remarkable for diffuse left lower extremity tenderness, pitting edema, and blanching erythema. Dorsalis pedis pulses were 2+ bilaterally, sensation was grossly intact, and there were no motor deficits noted. Homan’s sign was negative. Capillary refill was less than two seconds. She was afebrile and normotensive. The remainder of the physical exam was noncontributory. Initial lab work revealed an elevated fibrinogen at 486 mg/dL (normal range: 200-400 mg/dL) in the setting of normal prothrombin time (PT) and activated partial thromboplastin time (aPTT) values, suggestive of nonspecific inflammation. All other lab findings were within normal range. 

**Figure 2:**
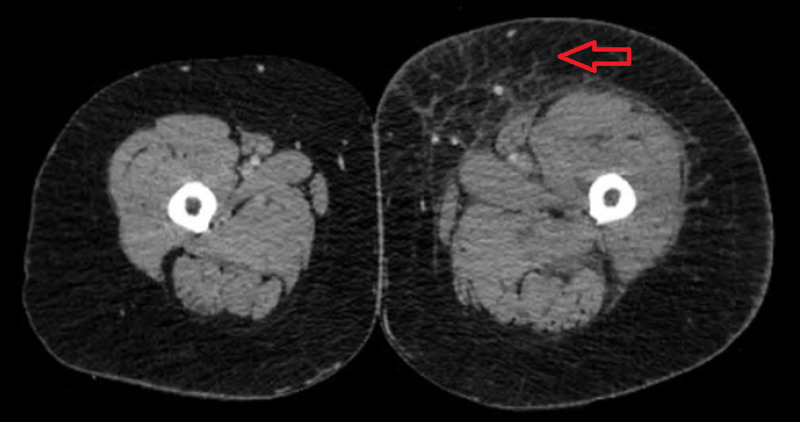
Transverse CT scan of bilateral lower extremities.

Clinical presentation was concerning for phlegmasia cerulea dolens. As such, a lower extremity ultrasound was bypassed in favor of a CT scan of bilateral lower extremities with IV contrast and venous runoff to evaluate for proximal venous obstruction. Imaging revealed distension of the left common iliac, external iliac, and left common femoral veins with inflammatory stranding, likely secondary to stenosis of the left common iliac vein just posterior to the right common iliac artery (Figure 2). This was deemed to be consistent with a May-Thurner abnormality.

**Figure 3:**
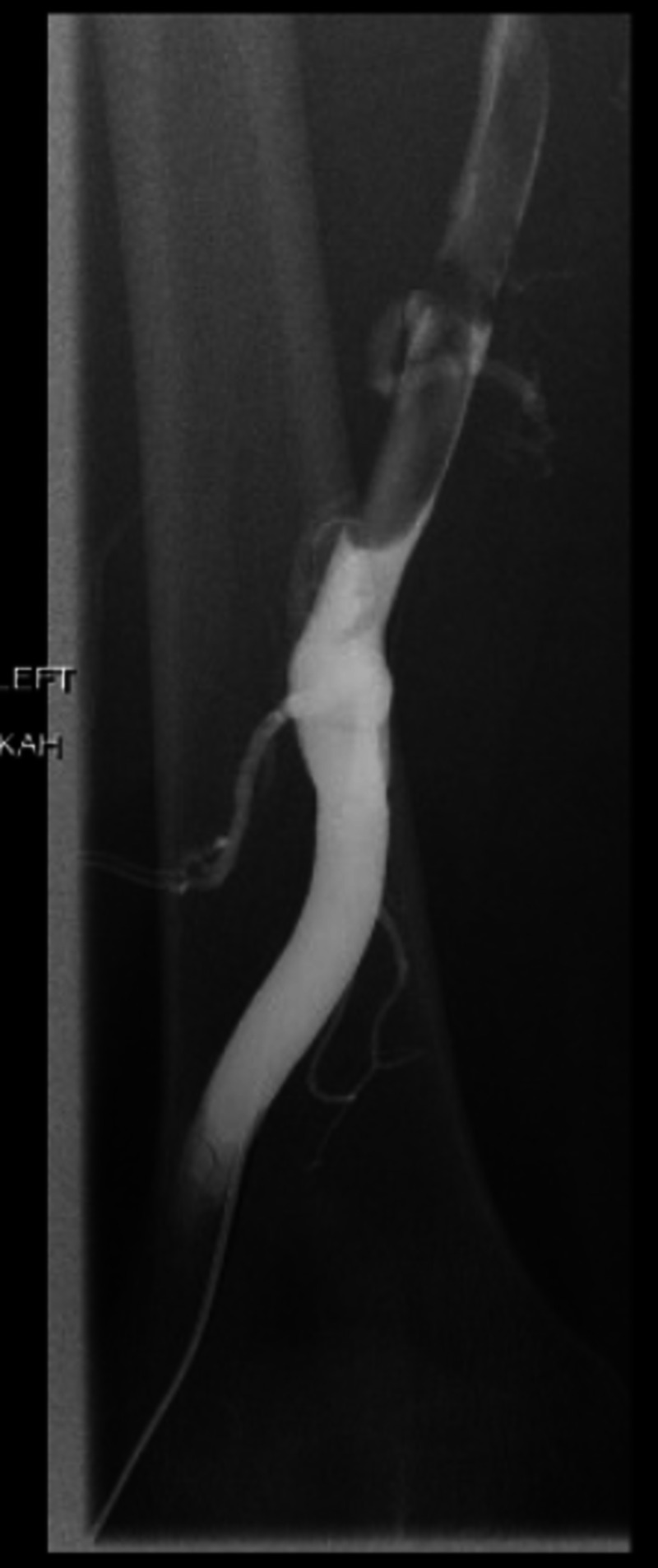
Left lower extremity venogram revealing near-occlusive thrombosis.

Interventional radiology was consulted, and they recommended catheter-directed tissue plasminogen activator (tPA) infusion followed by subsequent venous stenting. The patient was admitted to the ICU for the procedure. She received local anesthesia and fentanyl. A left lower extremity venogram was ordered and revealed near occlusive thrombosis of the left superficial femoral, common femoral, and iliac veins (Figure 3). Angioplasty was performed to create a channel with a 7 mm x 80 mm Mustang balloon and a 40 cm infusion length catheter was placed. Some 0.05 mg/mL alteplase was locally infused at a rate of 1 mg/h by way of catheter placement. The patient tolerated the procedure well and was transferred back to the ICU in stable condition.

**Figure 4:**
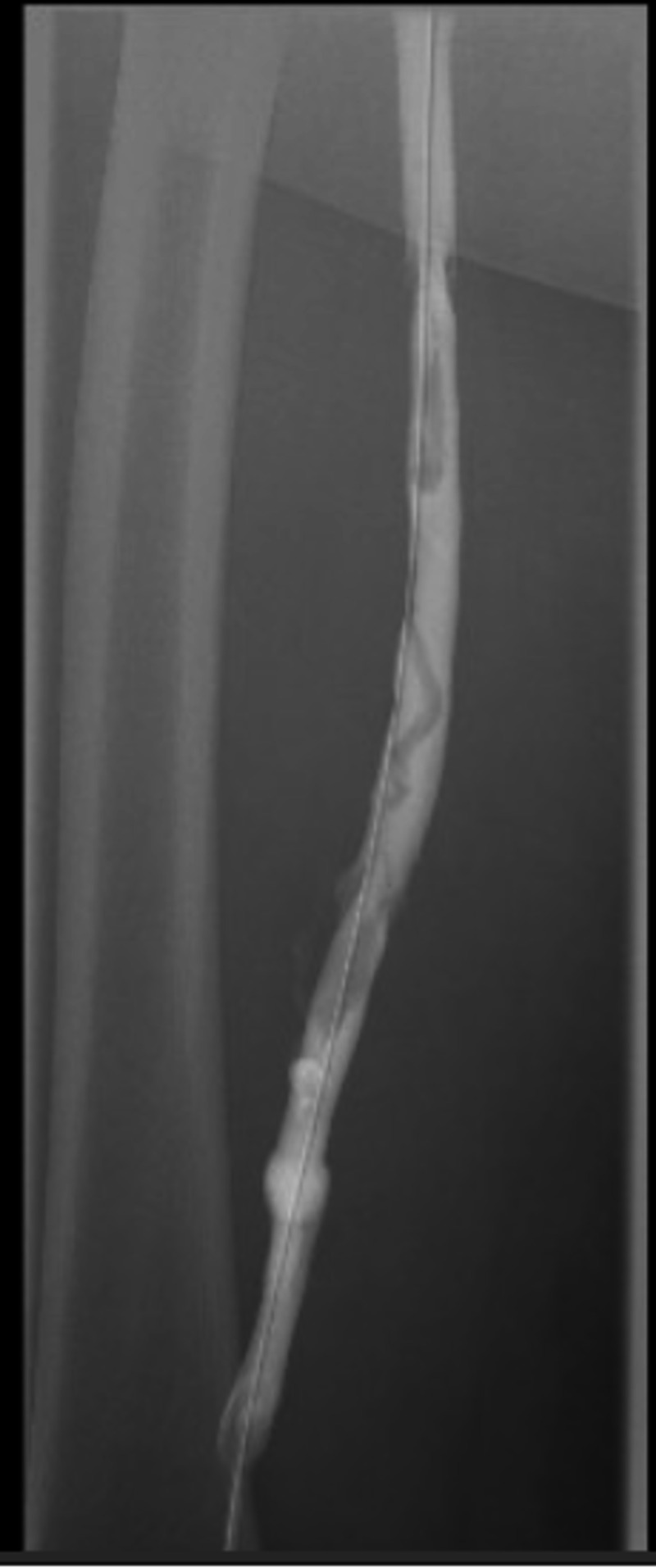
Left lower extremity venogram after pharmacologic thrombolysis.

The following day, a repeat venogram revealed improved blood flow in the femoral vein with mild to moderate residual thrombus (Figure 4). Large residual thrombus was noted in the left common iliac vein with persistent stenosis of the left common iliac vein. Repeat angioplasty using a 12 mm x 40 mm Mustang balloon was performed along with mechanical thrombectomy using an AngioJet device, but this was unsuccessful. A 5 French 20 cm infusion length catheter was placed in the left common iliac vein for further pharmacologic thrombolysis. 

**Figure 5:**
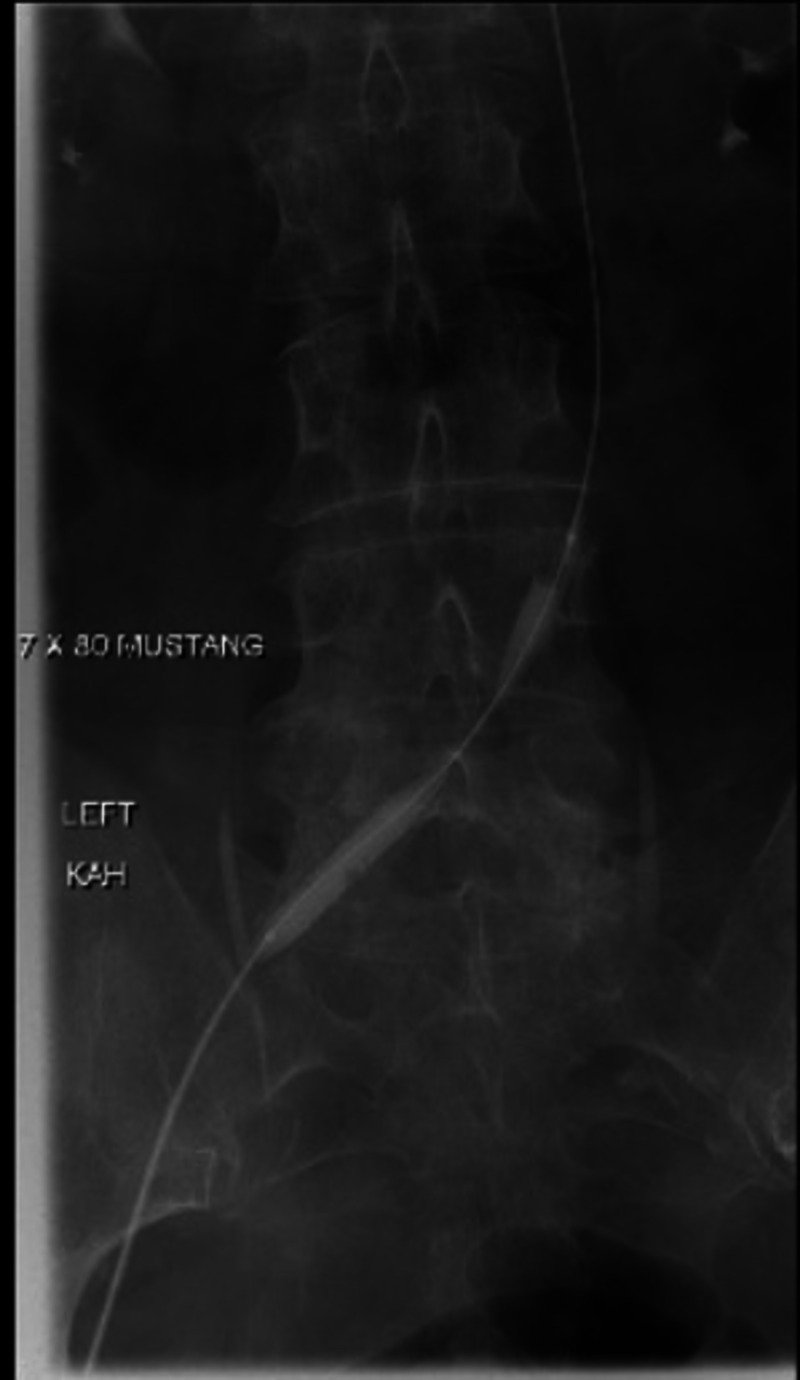
Left lower extremity venogram revealing dissolution of thrombus but severe stenosis of the left common iliac vein.

**Figure 6:**
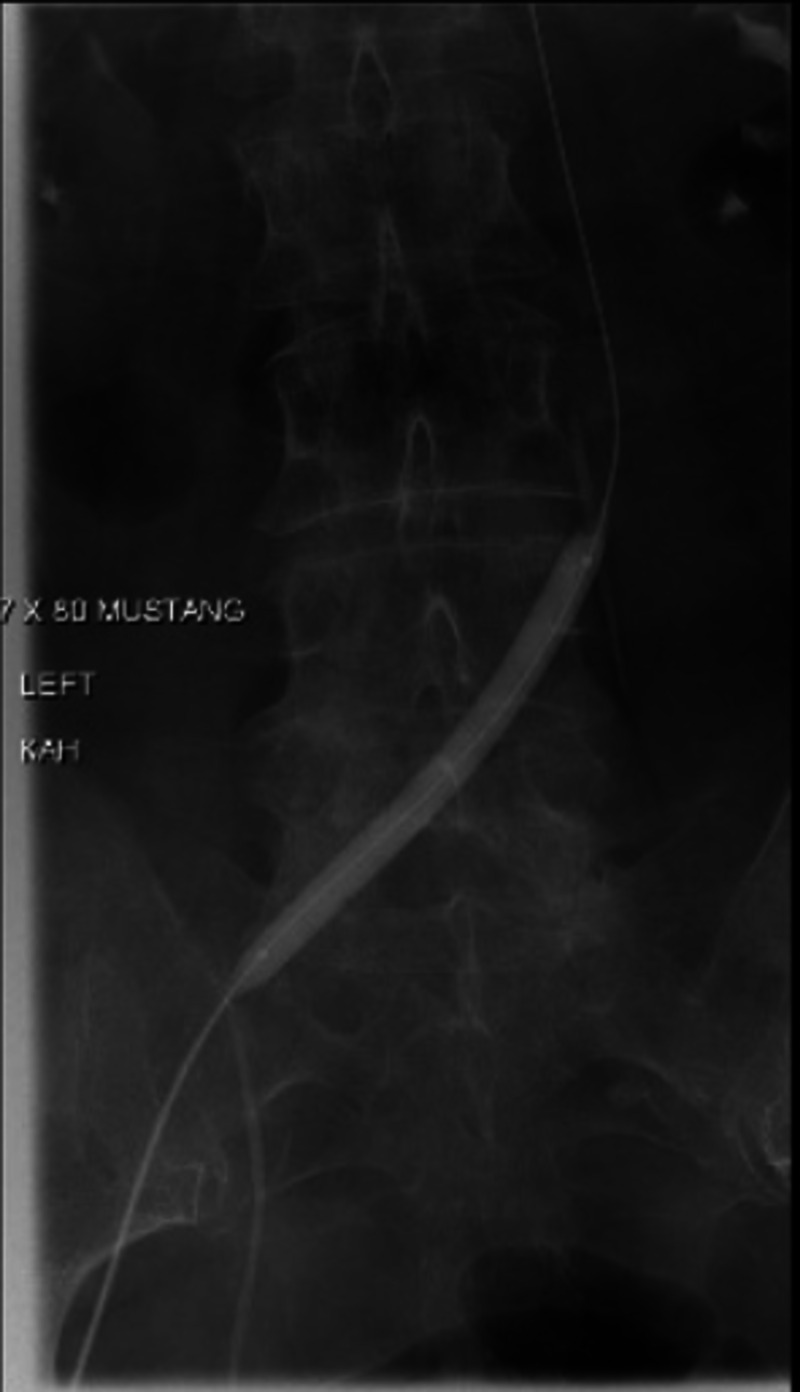
Left lower extremity venogram after endovascular stenting of the left common iliac vein.

The patient underwent a third venogram the next day that revealed dissolution of the thrombus but severe stenosis of the left common iliac vein and left external iliac vein (Figure 5). Stenting of the left common iliac vein was performed using an 18 mm x 60 mm Wallstent. Post-stent angioplasty was performed using a 16 mm x 40 mm Atlas balloon. A second Wallstent stent was placed in the left external iliac vein. Post-stent venogram revealed markedly improved blood flow through the left common iliac vein into the inferior vena cava (Figure 6). The patient tolerated the procedure well again with no complications.

The patient relayed improvement in her left lower extremity pain and swelling post-procedure. She was subsequently transferred out of the ICU and deemed stable for discharge on eliquis 5 mg twice a day for six months.

## Discussion

May-Thurner syndrome typically manifests secondary to extrinsic venous compression of the iliocaval veins by the anterior arterial system against the posterior lumbar vertebrae. The most common anatomic manifestation is secondary to compression of the left common iliac artery by the right common iliac artery. Other anatomic variants have been described, including extrinsic compression of the left common iliac vein by a distended bladder, endometriosis, a tortuous left common iliac artery, right common iliac artery aneurysm, and right internal iliac artery aneurysm [4]. Compression of the distal inferior vena cava by the right or left common iliac artery as well as compression of the right common iliac vein have also been observed [4].

Repeated pulsations of the right common iliac artery overriding the left common iliac vein stimulates intravascular fibrosis which subsequently leads to a variable degree of venous obstruction. May and Thurner initially noted this phenomenon and termed these fibrotic changes “venous spurs [2].” While these venous spurs reduce the intraluminal diameter of the vein and directly contribute to outflow obstruction, they also induce hemodynamic changes by disrupting laminar blood flow. These pulsations may also cause mild, chronic endothelial trauma which can predispose patients to thrombosis as well. Given our patient had a history of smoking along with current use of E-cigarettes, she met all three elements of Virchow’s triad (venous stasis, hypercoagulability, and endothelial injury).

While most patients with MTS are asymptomatic, patients can develop symptoms consistent with venous hypertension. These include acute extremity pain, swelling, venous claudication, ulceration, skin discoloration, chronic venous insufficiency, DVT, and post-thrombotic syndrome in select patients [2]. The initial evaluation of the patient with the aforementioned lower extremity symptoms involves determining the clinical likelihood of DVT using the Wells criteria. Those with a moderate to high pretest probability should undergo a venous duplex ultrasound, which should demonstrate narrowed iliac veins and absence of variation of flow [5]. However, our patient had extensive discoloration of the left lower extremity up to the groin that was concerning for phlegmasia cerulea dolens. The treatment team opted to proceed with noninvasive imaging in the form of a CT venogram to further evaluate for sources of proximal obstruction. According to Birn and Vedantham, clinical features that warrant further investigation for a May-Thurner diagnosis include pain/swelling of the entire limb with or without visible varicosities, dominance of venous claudication, persistent symptoms in spite of treatment of DVT, recurrent proximal DVTs, and stigmata of post-thrombotic syndrome [6]. Both CT and MR venography are highly sensitive for identifying and estimating stenosis, while also being able to investigate extrinsic sources of compression [7]. The CT venogram in our patient revealed proximal stenosis of the left common iliac vein by the right common iliac artery, a finding consistent with a May-Thurner abnormality.

Invasive imaging modalities include catheter-based venography (CBV) and intravascular ultrasound (IVUS) [2]. CBV is particularly effective when transvenous pressure measurements are added and is often considered the gold-standard test to diagnose MTS [2]. Pressure measurements obtained from both iliac veins can be obtained at rest and compared. An iliac vein pressure gradient of 2 mmHg or greater is suggestive of venous outflow obstruction [8]. As per Mickley et al. a pre-stenotic to post-stenotic peak vein velocity ratio of 2.5 is also effective in detecting a 50% stenosis of the affected vessel [9]. IVUS is beneficial in that it can capture the morphology of a venous spur while maintaining a sensitivity and specificity of 98% for detecting venous stenosis [9]. It can also be used in determining vessel diameter, aiding in stent placement, and estimating increased cross-sectional diameter status-post stent placement [2].

The general approach to treatment of a patient with MTS depends on the severity of symptoms, the presence of DVT, and the presence of post-thrombotic syndrome. Post-thrombotic syndrome refers to the presence of signs and symptoms consistent with chronic venous insufficiency after a DVT [10]. Roumen-Klappe et al. found the incidence of post-thrombotic syndrome after an acute DVT to be as high as 49% after one year, 55% after two years, and 56% after six years [10]. Furthermore, patients with extensive proximal (femoral or iliac vein) thrombosis had significantly worse post-thrombotic syndrome severity than those with distal or popliteal DVT as well as double the risk. Given the predisposition many patients with MTS have to develop proximal thrombosis, effective treatment geared towards the resolution of the clot as well as the prevention of post-thrombotic syndrome becomes paramount. In the absence of DVT, patients with minimal to mild symptoms receive conservative treatment through the use of compression stockings [2].

In symptomatic nonthrombotic MTS, stent placement can be used to reduce moderate to severe symptoms of venous hypertension. Neglén et al. examined 982 patients and found significantly higher stent patency in patients with nonthrombotic stigmata compared to patients with post-thrombotic syndrome [11]. Primary patency rates were 79% in nonthrombotic vascular lesions compared to 57% in patients with a history of DVT [11]. Secondary patency rates were also examined and were 100% in nonthrombotic vascular lesions versus 86% in patients with a history of DVT [11]. In addition, these patients often do not require anticoagulation as they are “relatively non-thrombotic” [6].

In symptomatic MTS with acute thrombosis, anticoagulative therapy remains the mainstay of treatment. For patients with recurrent DVTs, catheter-directed thrombolysis and surgical venous thrombectomy (in those with contraindications to thrombolysis) with subsequent stent placement has been associated with low rates of postoperative re-thrombosis as seen in Mickley et al.’s retrospective study [9]. In contrast, the nonstented control group had postoperative early re-thrombosis rates as high as 73% (p<0.01) [9]. Furthermore, in a 473-patient multicenter prospective registry of DVT patients that had catheter-directed thrombolysis, patients who underwent subsequent stent placement were found to have a significantly greater one-year venous patency than those who did not undergo stent placement [12]. These findings underscore the importance of endovascular stent placement in stenosed vessels post-thrombectomy.

The efficacy of percutaneous endovenous intervention (PEVI) with anticoagulation versus anticoagulation alone in preventing venous thromboembolism and post-thrombotic syndrome was examined in Sharifi et al.’s TORPEDO trial [13]. Anticoagulation consisted of IV unfractionated heparin or a subcutaneous low molecular weight heparin with warfarin while PEVI consisted of one or more of a combination of thrombectomy, balloon venoplasty, stenting, or local low dose thrombolytic therapy [13]. At six months follow-up, recurrent VTE was seen in 2.3% of the PEVI + anticoagulation group versus 14.8% in the control group (p=0.003). Post-thrombotic syndrome was noted in 3.4% of the PEVI + anticoagulation group versus 27.2% in the anticoagulation group alone (p<0.001) [13]. This study illustrates that while anticoagulation is essential in treating patients with acute DVT, endovascular techniques have become a vital adjunct in the treatment algorithm. Concordant findings were noted in a Norwegian study conducted by Enden et al. They found a statistically significant increase in iliofemoral patency at six months in 65.9% of patients receiving catheter-directed thrombolysis versus 47.4% of patients receiving conventional anticoagulation (p=0.012) [14]. At 24 months, 41.1% of patients on catheter-directed thrombolysis exhibited post-thrombotic syndrome compared to 55% in the control group (p=0.047) [14]. Interestingly, the results of the ATTRACT trial contradict the results of the aforementioned studies and showed that pharmacomechanical catheter-directed thrombolysis did not lower the risk of post-thrombotic syndrome but did reduce the severity of post-thrombotic syndrome [15]. Catheter-directed thrombolysis was also found to be associated with an increased risk of bleeding [15]. Other complications of endovascular therapy include thrombosis of the contralateral vein, rupture of the iliac vein, stent displacement, and erosion of the stent into the overlying artery [2].

Historically, surgical venous bypass has been used to provide symptom relief to patients with post-thrombotic syndrome associated with iliac vein stenosis. However, this procedure requires specialized venous surgeons and are becoming rarer in patients with no contraindications to thrombolysis [2]. Surgical bypass options include cross-femoral venous bypass, femerofemoral bypass, ilioiliac prosthetic bypass, and femerocaval and aortic elevation [16]. Jost et al. found venous reconstructions for iliofemoral or IVC obstruction had three-year patency rates of 62% with cross-femoral venous bypass with autologous saphenous vein having the highest long-term patency at 83% [16]. Common femoral endovenectomy with endoluminal iliocaval recanalization was studied by Vogel et al. in 2010 and was found to improve objective outcome measures with chronic post-thrombotic obstruction [17]. This finding was echoed by Comerota et al.’s research on the treatment of a longstanding venous ulcer in a patient with post-thrombotic occlusion of the iliofemoral venous segments using operative endovenectomy and patch venoplasty [18]. Nonetheless, open surgery is indicated only in the setting of failed endovascular therapy or contraindications to thrombolysis given the morbidity and overall worse results with open surgical intervention.

Stent recanalization in patients with post-thrombotic syndrome with iliac vein occlusion has been examined in several studies and nearly universally found to be beneficial in the treatment of venous outflow obstruction. One such study conducted by Raju and Neglén found that relief of pain and swelling at three years was 79% and 66% respectively [19]. Healing of venous ulcers at 33 months was 56% [19]. Cumulative secondary stent patency rate at four years was 66% [19]. Nayak et al.’s retrospective study found that the proportion of limbs with pain and swelling after endovascular intervention was as low as 35% and 50% (compared to 82.5% and 90.0% before, p<0.01) [20]. Typically, a venogram or IVUS is performed to identify the stenosed segment, followed by guidewire recanalization [7]. Pre-dilatation with an angioplasty balloon is then performed, followed by stent placement ranging from 12 to 18 mm in diameter [7]. This was the exact way interventional radiology proceeded to treat the patient discussed above and serves to highlight the crucial role endovascular stent placement has come to play in the resolution of venous hypertension. 

## Conclusions

May-Thurner syndrome is an underdiagnosed condition characterized by external compression of iliocaval vascular structures. While many patients are asymptomatic due to the variable degree of extrinsic compression, those with symptoms often exhibit signs of venous hypertension secondary to venous outflow obstruction. Those with MTS are at higher risk for venous ulcers, claudication, swelling, pain, skin discoloration, acute DVT, and reduced quality of life. The presence of acute DVT also predisposes patients to developing the post-thrombotic syndrome, leading to a more chronic form of venous insufficiency. As a result, timely recognition and diagnosis followed by appropriate treatment becomes paramount.

This case underscores the role endovascular intervention plays in the treatment of not only MTS, but most forms of venous outflow obstruction. While surgical intervention and anticoagulation used to be the mainstays of treatment, the literature suggests they are better suited as adjuncts in patients with no contraindications to pharmacomechanical thrombolysis and stent placement. Our onsite interventional radiology team shared this sentiment and was successfully able to perform catheter-directed thrombolysis and stent placement in the left common iliac and left external iliac veins. The patient’s symptoms improved with restored vessel patency, and she was discharged shortly thereafter with anticoagulative therapy.

## References

[1] Thurner J (1957). The cause of the predominantly sinistral occurrence of thrombosis of the pelvic veins - R. May, J. Thurner, 1957. SAGE J.

[2] Mousa Mousa, Albier Y (2020). May-Thurner Syndrome. UpToDate, July.

[3] Cockett FB, Thomas ML, Negus D (1967). Iliac vein compression -- its relation to iliofemoral thrombosis and the post-thrombotic syndrome. Br Med J.

[4] Kibbe MR, Ujiki M, Goodwin AL (2004). Iliac vein compression in an asymptomatic patient population. J Vasc Surg.

[5] Lensing AW, Prandoni P, Brandjes D (1989). Detection of deep-vein thrombosis by real-time B-mode ultrasonography. N Engl J Med.

[6] Birn J, Vedantham S (2015). May-Thurner syndrome and other obstructive iliac vein lesions: meaning, myth, and mystery. Vasc Med (Lond, Engl).

[7] Wolpert LM, Rahmani O, Stein B, Gallagher JJ, Drezner AD (2002). Magnetic resonance venography in the diagnosis and management of May-Thurner syndrome. Vasc Endovasc Surg.

[8] Ferris EJ, Lim WN, Smith PL, Casali R (1983). May-Thurner syndrome. Radiology.

[9] Mickley V, Schwagierek R, Rilinger N, Gorich J, Sunder-Plassmann L (1998). Left iliac venous thrombosis caused by venous spur: treatment with thrombectomy and stent implantation. J Vasc Surg.

[10] Roumen-Klappe EM, Heijer M, Janssen MCH (2005). The post-thrombotic syndrome: incidence and prognostic value of non-invasive venous examinations in a six-year follow-up study. Thromb Haemost.

[11] Neglén P, Hollis KC, Olivier J, Raju S (2007). Stenting of the venous outflow in chronic venous disease: long-term stent-related outcome, clinical, and hemodynamic result. J Vasc Surg.

[12] Mewissen MW, Seabrook GR, Meissner MH, Cynamon J, Labropoulos N, Haughton SH (1999). Catheter-directed thrombolysis for lower extremity deep venous thrombosis: report of a national multicenter registry. Radiology.

[13] Sharifi M, Mehdipour M, Bay C, Smith G, Sharifi J (2010). Endovenous therapy for deep venous thrombosis: the TORPEDO trial. Catheter Cardiovasc Interv.

[14] Enden T, Haig Y, Klow N (2012). Long-term outcome after additional catheter-directed thrombolysis versus standard treatment for acute iliofemoral deep vein thrombosis (the CaVenT Study): a randomised controlled trial. Lancet (London, England).

[15] Vedantham S, Goldhaber SZ, Julian JA (2017). Pharmacomechanical catheter-directed thrombolysis for deep-vein thrombosis. N Engl J Med.

[16] Jost CJ, Gloviczki P, Cherry Jr. KJ (2001). Surgical reconstruction of iliofemoral veins and the inferior vena cava for nonmalignant occlusive disease. J Vasc Surg.

[17] Vogel D, Comerota AJ, Al-Jabouri M, Assi ZI (2012). Common femoral endovenectomy with iliocaval endoluminal recanalization improves symptoms and quality of life in patients with postthrombotic iliofemoral obstruction. J Vasc Surg.

[18] Comerota AJ, Grewal NK, Thakur S, Assi Z (2010). Endovenectomy of the common femoral vein and intraoperative iliac vein recanalization for chronic iliofemoral venous occlusion. J Vasc Surg.

[19] Seshadri R, Neglén P (2009). Percutaneous recanalization of total occlusions of the iliac vein. J Vasc Surg.

[20] Nayak L, Hildebolt CF, Vedantham S (2012). Postthrombotic syndrome: feasibility of a strategy of imaging-guided endovascular intervention. J Vasc Intervent Radiol.

